# Spatial heterogeneity and correlates of child malnutrition in districts of India

**DOI:** 10.1186/s12889-018-5873-z

**Published:** 2018-08-17

**Authors:** Junaid Khan, Sanjay K Mohanty

**Affiliations:** 1 International Institute for Population Sciences, Govandi Station Road, Deonar, Mumbai, 400088 India; 2 Department of Fertility Studies, International Institute for Population Sciences, Govandi Station Road, Deonar, Mumbai, 400088 India

**Keywords:** Malnutrition, Stunting, Wasting, Underweight, Spatial heterogeneity, India

## Abstract

**Background:**

Despite sustained economic growth and reduction in money metric poverty in last two decades, prevalence of malnutrition remained high in India. During 1992–2016, the prevalence of underweight among children had declined from 53% to 36%, stunting had declined from 52% to 38% while that of wasting had increased from 17% to 21% in India. The national average in the level of malnutrition conceals large variation across districts of India. Using data from the recent round of National Family Health Survey (NFHS), 2015–16 this paper examined the spatial heterogeneity and meso-scale correlates of child malnutrition across 640 districts of India.

**Methods:**

Moran’s *I* statistics and bivariate LISA maps were used to understand spatial dependence and clustering of child malnutrition. Multiple regression, spatial lag and error models were used to examine the correlates of malnutrition. Poverty, body mass index (BMI) of mother, breastfeeding practices, full immunization, institutional births, improved sanitation and electrification in the household were used as meso scale correlates of malnutrition.

**Results:**

The univariate Moran’s *I* statistics was 0.65, 0.51 and 0.74 for stunting, wasting and underweight respectively suggesting spatial heterogeneity of malnutrition in India. Bivariate Moran’s *I* statistics of stunting with BMI of mother was 0.52, 0.46 with poverty and − 0.52 with sanitation. The pattern was similar with respect to wasting and underweight suggesting spatial clustering of malnutrition against the meso scale correlates in the geographical hotspots of India. Results of spatial error model suggested that the coefficient of BMI of mother and poverty of household were strong and significant predictors of stunting, wasting and underweight. The coefficient of BMI in spatial error model was largest found for underweight (β = 0.38, 95% CI: 0.29–0.48) followed by stunting (β = 0.23, 95% CI: 0.14–0.33) and wasting (β = 0.11, 95% CI: 0.01–0.22). Women’s educational attainment and breastfeeding practices were also found significant for stunting and underweight.

**Conclusion:**

Malnutrition across the districts of India is spatially clustered. Reduction of poverty, improving women’s education and health, sanitation and child feeding knowledge can reduce the prevalence of malnutrition across India. Multisectoral and targeted intervention in the geographical hotspots of malnutrition can reduce malnutrition in India.

**Electronic supplementary material:**

The online version of this article (10.1186/s12889-018-5873-z) contains supplementary material, which is available to authorized users.

## Background

Reduction in malnutrition and poverty is the primary agenda among public health professionals, planners and policy makers at global, national and regional level. Though nutritional deficiency affects all age groups, children under five-year age group are at higher risk. Globally, 156 million children under five years of age are stunted, 93 million are underweight and 50 million are wasted [1]. The global efforts on reduction of child malnutrition began with the Copenhagen Consensus and continued through the millennium development goals (MDGs) and Sustainable Development Goals (SDGs). Goal 1 of MDGs aimed at eradication of poverty and hunger and targeted reducing the number of underweight children by half by 2015 from 1990 level. Goal 2 of SDGs aimed to end hunger and all forms of malnutrition by 2030. Despite concerted efforts globally and nationally, the prevalence of malnutrition remained high in developing countries, particularly in the South Asian Regions.

Malnutrition is the primary cause of immuno-deficiency among the infants and the five infectious diseases (pneumonia, diarrhoea, malaria, measles, and AIDS) contribute to the half of all deaths in children aged less than five years and are directly associated with one or other measures of malnutrition [2–4]. About half of the infant deaths is due to malnutrition and it is the single largest factor contributing to the global burden of disease [5, 6]. And the risk of dying from diseases like diarrhoea, acute respiratory infections, malaria and measles increases with increased malnutrition among the children [7, 8]. The hospital based studies also suggest that children’s nutritional status at the time of hospital admission is significantly associated with the risk of dying from Malaria and Measles [8, 9]. The low birth weight of children is associated with morbidity in the adulthood and particularly obesity, cardio vascular diseases and type-2 diabetes [10–13]. Additionally, preconceptional diet pattern of the mothers can increase the chance of birth weight of the children [13].

Malnutrition is primarily caused due to the immediate causes (inadequate dietary intake, lack of care and disease), underlying causes (inadequate access to food, improper care of mothers and children and scarcity of health services) and the basic causes (an unhealthy environment and inadequate education, formal and non-formal institutions, political and ideological superstructures, economic structures and a lack of potential resource) [14–17]. The underlying and the immediate causes of malnutrition are due to inadequate food intake and studies suggest a strong and positive association between consumption expenditure of the household and child’s malnutrition [18–21]. A large number of studies examined the other economic gradient of nutrition in developing countries suggesting lack of resources and food unavailability due to poverty causes pathways and increases the level of malnutrition in the population [22–26]. The poverty condition in a household creates the pathways to malnourishment among the children. Similarly, improved sanitation in the households minimise the chance of infection among the children and studies found positive association between household level sanitation and linear growth among the children [27]. The maternal health, particularly, the Low BMI (less than 18.5 kg/m^2^) of mother is consistently found a high risk factor of poor intrauterine growth and low birth weight may lead to malnourished children [28, 29]. Parental educational attainments are also significantly associated with lower childhood malnutrition [30].

During last two decade, India has experienced sustained economic growth (over 5% growth in GDP) and reduced the poverty level by half (from 50% in 1993–94 to 22% by 2011–12) but reduction in stunting, underweight and wasting has not been observed in the same scale [31]. Evidences suggest that the level of stunting children has declined from 52% in 1992–93 to 38% by 2015–16 but the prevalence of wasting had increased from 17% to 21% during this period. And in 2016, India accounted 62 million of stunted children, 40% of the global share of stunting [32].

### Scope of study

The aim of this study is to understand the spatial heterogeneity and meso-scale correlates of malnutrition across the districts of India. The study has been conceptualised with the following rationale.

First, most of the studies for India examined the levels of malnutrition and explored the social and demographic correlates of malnutrition at individual and household level. However, the districts of India are large and exhibit enormous variation in socio-demographic and economic indicators. And very little is known about the spatial heterogeneity of malnutrition, particularly poverty and the associated nexus with malnutrition at a meso scale, i.e., in the districts of India. Second, geographical location has a large impact on the distal determinants of nutrition which directly or indirectly controls the availability and accessibility of foods mainly due to agricultural patterns and yields. Third, a district level analysis of malnutrition is very much needed considering the spatial occurrence of poverty. Fourth, India ranked 97th globally (118 countries) in the hunger index and the hunger index shows huge variation across the states of India [33]. Studies also suggest that poverty-nutrition trap do exist in India [34].

### Data and methods

#### Data

Data from the recently conducted National Family Health Survey round four (NFHS-4), 2015–16 is used in the analysis. NFHS 4 provides comprehensive information on fertility, mortality, maternal health care and child health including child nutrition for the states and districts of India. NFHS-4 covered a nationally representative sample of 601,509 households, 699,686 women aged 15–49 years and 103,525 men aged 15–54 years in India [35]. The average number of households surveyed in each district was about 940. District is the unit of analysis in this study. A district level data file is prepared on key socio-demographic, economic and nutritional indicators. The district level estimates are available for public use on (http://rchiips.org/NFHS/districtfactsheet_NFHS-4.shtml). The unit level data is available from Demographic Health Survey (DHS) data repository and could be accessed upon a data request through www.dhsprogram.com/data/ [35–37]. The analyses are based upon 640 districts of India. The socio-economic and demographic estimates of districts of India were also compiled from NFHS 4. District level estimates on consumption poverty (poverty head count ratio) were taken from published sources [38]. By malnutrition, we referred to stunting, wasting and underweight throughout the paper.

### Outcome variables

Three anthropometric indicators of nutritional status of children namely stunting (height-for-age), underweight (weight-for- age) and wasted (weight-for-height) are used as the dependent variables in this study. These three measures are the standardized measures of nutritional status of children and commonly used in literature [39, 40]. While stunting measures the chronic nutritional deprivation, underweight indicates an acute and chronic form of undernourishment and wasting suggests an acute nutritional deficiency among the children [41].

### Independent variables

A set of proximate and distal determinants of malnutrition within a meso scale framework were used based on prior literature and data availability [42]. Child feeding practices, full immunization, improved sanitation, electrification and poverty level of the household were included as proximate determinants. Similarly, women’s education, body mass index, institutional delivery were used as distal determinants. The utility of including these variables in the analyses has been reviewed in the previous section.

## Methods

We have used descriptive statistics, estimated univariate and biravriate Moran’s *I* statistics and a set of regression models in the analyses. Differentials in malnutrition by its correlates were presented using bivariate analyses. Univariate LISA and bivariate LISA maps were utilized in this study to identify the spatial clusters. Univariate LISA map provided the geographical clustering of different variables used in this study while Bivariate LISA measured the correlation between the independent and the weighted average of the dependent variable in a particular location. To understand the significant correlates of malnutrition, a set of regression models were used to give the best fit of the data and to understand the association of malnutrition and its correlates. At first, we used the Ordinary Least Square (OLS) regression with each of the outcome variables and estimated the extent of spatial autocorrelation in the error term and the corresponding Moran’s I statistic. Since the OLS confirmed spatial autocorrelation in its error term for all three outcome variables, we further estimated spatial lag model (SLM) and Spatial Error Model (SEM). The underlying assumption of a spatial lag model is that the observations of the dependent variable are affected in the neighbourhood areas whereas the spatial error model is used to consider the effect of those variables which are not present in the regression model but have an effect on the outcome variable. The basic difference between the two models is that the spatial lag model unlike spatial error model does not consider the spatial dependence in the error term. Diagnostics tests for spatial dependence were carried out, and the value of Lagrange Multiplier was found significant in both the models(*p* < 0.0001) and next we compared the Akaike Information criterion (AIC) value for both the models to know the best spatial fit. This spatial models examined the non linear relationship between the correlates and malnutrition at a meso scale controlling other demographic and distal covariates subject to the spatial structure of the data [43]. Each of the models is specified below.

The multiple linear regression model is given as;

1 $$ {\mathrm{Y}}_{\mathrm{i}}=\upalpha +{\upbeta}_1{\mathrm{BMI}}_{\mathrm{i}}+{\upbeta}_2{\mathrm{CPOV}}_{\mathrm{i}}+{\upbeta}_3{\mathrm{WEDU}}_{\mathrm{i}}+{\upbeta}_4{\mathrm{ID}}_{\mathrm{i}}+{\upbeta}_5{\mathrm{FI}}_{\mathrm{i}}+{\upbeta}_6{\mathrm{CFP}}_{\mathrm{i}}+{\upbeta}_7{\mathrm{IS}}_{\mathrm{i}}+{\upbeta}_8{\mathrm{ELECT}}_{\mathrm{i}} $$

Where Y_i_ denotes the proportion of children malnourished in the i-th district;

α is the intercept.

β _j_ denotes the regression coefficient for the jth variable where j = 1 (1) 8.

i (1, 2,…, 640) denotes the no of districts.

BMI denotes the percentage of women in the i-th district whose body mass index (BMI) is below 18.5 kg/m^2^, CPOV is the poverty head count ratio for the i-th district, WEDU is the district level proportion of women with 10 or more years of schooling, ID gives the district level percentage of institutional delivery, FI is the percentage of full immunization in the district; CFP is the percentage of children in the i-th district who are breastfed and received adequate diet; IS is the percentage of households with improved sanitation and ELECT denotes the percentage of households in the i-th district with electricity connection.

The spatial lag model assumes that the dependent variable in one area is affected by the dependent variable in the nearby. A typical spatial lag model can be written as follows:

2 $$ {Y}_i=\delta \sum \limits_{j\ne 1}{W}_{ij}{Y}_j+\beta {X}_j+{\varepsilon}_j $$

Here Y_i_ denotes the prevalence of malnutrition for the i-th district, δ is the spatial autoregressive coefficient, W_ij_ denotes the spatial weight of proximity between district i and j, Y_j_ is the prevalence of malnutrition in the j-th district, βj denotes the coefficient, X_j_ is the predictor variable and ε_j_ is the residual.

A Spatial Error Model (SEM) is expressed as follows:

3 $$ {Y}_i=\beta {X}_j+\lambda \sum \limits_{j\ne 1}{W}_{ij}{Y}_j{\varepsilon}_j+{\varepsilon}_i $$

Here Y_i_ denotes the prevalence of malnutrition for the i-th district, λ is the spatial autoregressive coefficient, W_ij_ denotes the spatial weight of proximity between district i and j, Y_j_ is the prevalence of malnutrition in the j-th district, βj denotes the coefficient, X_j_ is the predictor variable and *ε*_*i*_ is the residual.

ArcGIS version 10.1, GeoDa version 1.6.7 and STATA 12.0 are used for analyzing the data.

## Results

**Map 1:**
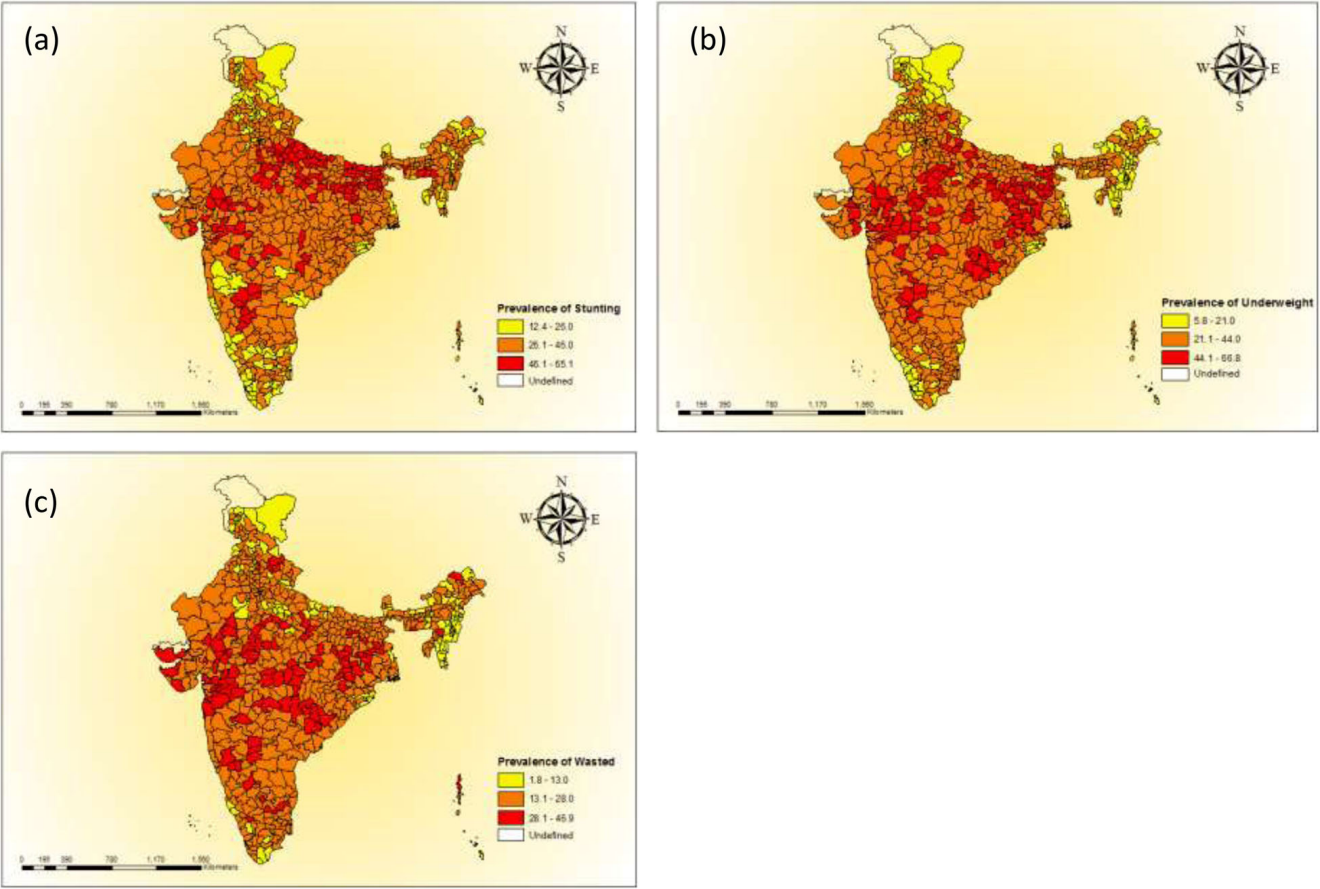
Maps of India showing the geographic distribution of the rates of malnutrition (**a**) stunting (**b**) underweight (**c**) wasting across districts of India, 2015-16. Source: Authors generated the maps using ArcGIS version 10.1

We begin the discussion by presenting the spatial pattern of stunting, underweight and wasting in districts of India (Map 1). The districts were classified into three broad categories; low, medium and high in each of the three indicators based on the mean and standard deviation. With respect to stunting, 110 districts were classified as high on malnourished children (stunting prevalence higher than 46%), 410 districts as medium (stunting prevalence in the range of 26.1–46%) and 120 districts as low (stunting prevalence of less than 26%). Similarly in case of underweight, about 117 districts were classified as high (underweight prevalence more than 44.1%), 407 districts in the medium category (underweight prevalence in the 21.1–44%) and 116 districts in the lowest category of underweight (underweight prevalence of less than 21.1%). Whereas, with respect to wasting 109 districts were classified in the category of highest wasted prevalence (more than 28%). A substantial number of districts with high prevalence of stunting and underweight are from the poorer states of Bihar, Uttar Pradesh, Madhya Pradesh and Rajasthan. Also some districts from the richer states of Maharashtra (For example, Yavatmal, Nandurbar and Parbhani districts) and Gujarat (Narmada, Sabar Kantha, The Dangs and Anand districts) had higher level of stunting (Additional file 1: Appendix 4). The stunting prevalence was as high as 65% in Bahraich district of Uttar Pradesh followed by Rampur district of the same state. Similarly, the level of stunting was lowest in Ernakulam district of Kerala. Additional file 1: Appendix 4–6 provides the list of districts with higher prevalence of stunting, underweight and wasted children respectively.

**Fig. 1:**
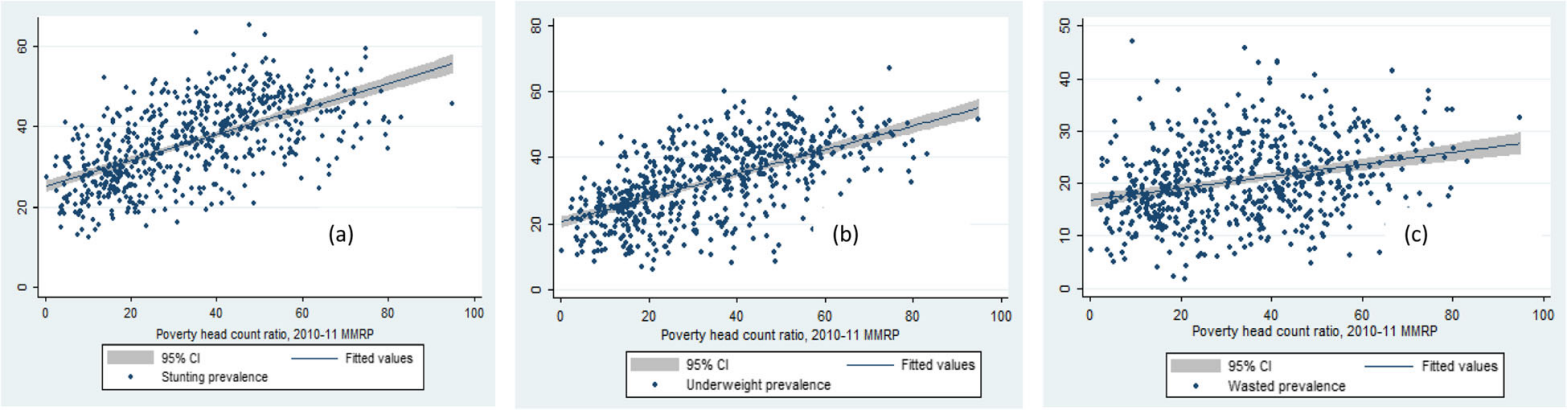
Graphs visualizing the scatter plots between PHCR and (**a**) stunting (**b**) underweight (**c**) wasting across districts of India, 2015–16. Source: Authors generated the figures

Table 1 presents the mean, standard deviation, coefficient of variation, minimum and maximum value of the variables used in the study. The distribution of the variables suggested wide variation in each of the dependent and the independent variables. The coefficient of variation for stunting was 27.5, 35.8 for underweight and 37.4 for wasted prevalence suggesting wide variation in the anthropometric indicators of malnutrition across India. Among the independent variables, a higher coverage of electrification and institutional births was observed across the districts. It was also observed that the coverage of exclusive breast feeding and immunisation is far from universal coverage in districts of India. The coefficient of variation was highest for breastfeeding followed by poverty and least for electricity connection. While some of the districts achieved universal electrification, institutional births and child immunisation, such coverage were also abysmally low in some other districts of India.

**Table 1:** Descriptive Statistics of the selected indicators/variables, India, 2015–16

Variables (district level percentages)	Mean	Standard Deviation	Coefficient of variation	Minimum	Maximum
Stunted	36	9.9	27.5	12.4	65.1
Underweight	32.7	11.7	35.8	5.8	66.9
Wasted	20.6	7.7	37.4	1.8	46.9
Women whose BMI is below 18.5 kg/m^2^	22.2	8.9	40.1	3.3	47.5
Poverty Head Count ratio	33.8	18.7	55.3	0.3	95
women with 10 or more years schooling	34.5	14.4	41.7	9	86.3
Institutional births	78.9	17.3	21.9	9.6	100
Children fully Immunized	62.2	17.3	27.8	7.1	100
Children breastfed & received adequate diet	9.7	7.6	78.4	0	39.5
Households with improved sanitation	48	22.6	47.1	6.9	99.5
Households with electricity	88	14.7	16.7	25.6	100
No of districts	640				

**Fig. 2:**
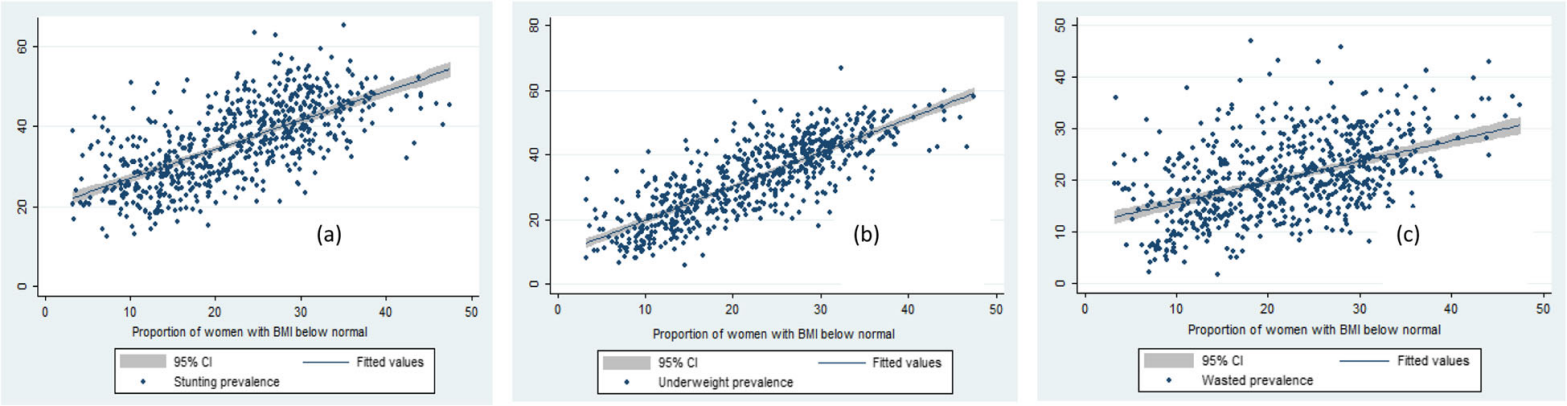
Graphs visualizing the scatter plots between BMI status of women and (**a**) stunting (**b**) underweight (**c**) wasting across districts of India, 2015–16. Source: Authors generated the figures

**Fig. 3:**
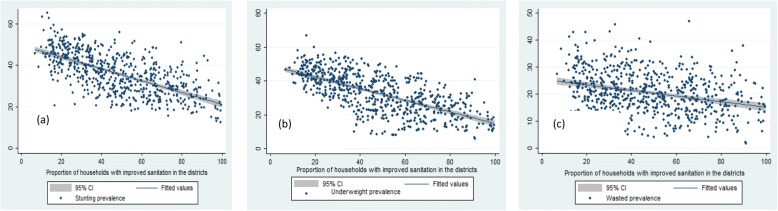
Graphs visualizing the scatter plots between sanitation condition and (**a**) stunting (**b**) wasting (**c**) underweight across districts of India, 2015–16. Source: Authors generated the figures

**Fig. 4:**
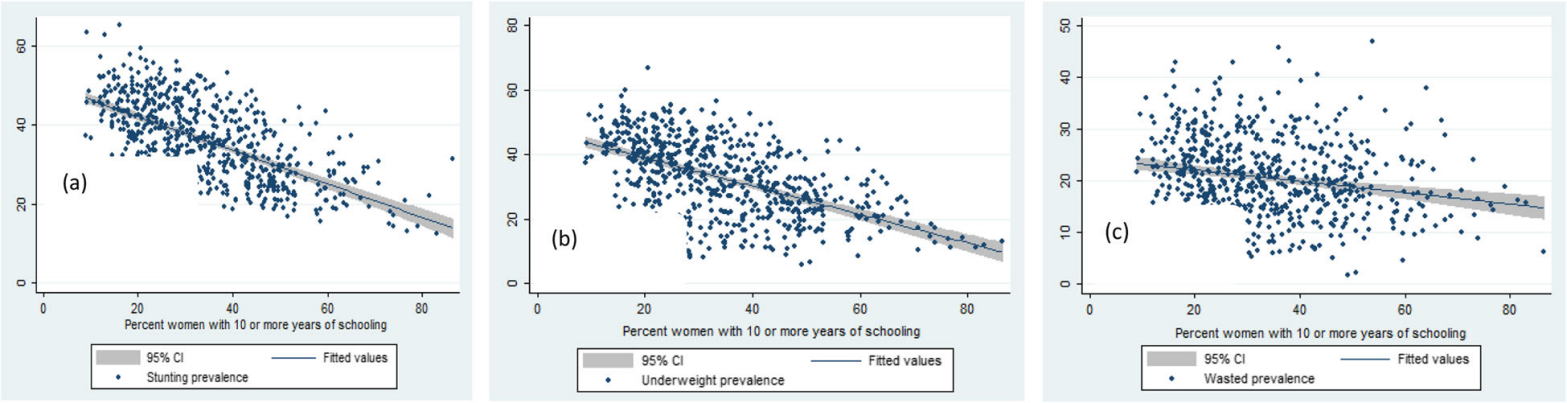
Graphs visualizing the scatter plots between women’s education and (**a**) stunting (**b**) underweight (**c**) wasting across districts of India, 2015–16. Source: Authors generated the figures

Table 2, Fig 1, 2, 3 and 4 presents the variation in stunting, underweight and wasted prevalence by the meso scale correlates in districts of India. Table 2 presents the mean estimates of the malnutrition indicators for each of the category of independent variables. Figure 1, presents the scatter plots of stunting, underweight and wasting by poverty across the districts of India. Districts with higher incidence of poverty showed higher prevalence in stunting, underweight and wasting. The average prevalence of stunting was 30% in districts with PHCR less than 30 compared to 44% in districts with PHCR more than 60 (Table 2). This pattern of poverty gradient also holds true for the prevalence of underweight and wasting as well. Considering maternal nutrition we found, districts where15% of the mothers were below the normal BMI (18.5 kg/m^2^), the average prevalence of stunting was 27.5% and it was found 44.5% in those districts where 30% of the mothers were undernourished (less than 18.5 kg/m^2^BMI). Figure 2 showed the scatteredness of malnutrition indicators by the women’s BMI across the districts. The scatter plot clearly depicted a positive slope of BMI with stunting and underweight. For example, districts where more than 30% of women had their BMI level below normal showed higher prevalence of malnutrition (45% stunting and underweight each and 26% wasted) compared to the rest of the districts (Table 2). Figure 3 presents the scatter plots between level of improved sanitation and malnutrition in districts of India. The scatter plots suggested a lower prevalence of malnutrition in districts where sanitation coverage was higher and a clear declining pattern of malnutrition was evident with increased level of improved sanitation condition. Like sanitation, educational status of the mothers (10 or more years of schooling) showed a negative association with the malnutrition in districts of India. Similarly, disparity in the coverage of full immunization and children’s feeding practices showed substantial variations in the level of malnutrition across the districts of India (Table 2).

**Table 2:** Average prevalence of malnutrition (percentage) by meso scale correlates across the districts in India, NFHS, 2015–16, India

District level Meso scale correlates (%)	Stunting	Underweight	Wasted
Women whose BMI is below 18.5 kg/m^2^			
< 15	27.5	20.2	16.0
15–30	36.9	34.1	21.0
> 30	44.5	44.8	25.5
Poverty Head Count ratio (%)			
< 30	30.2	26.4	18.7
30–60	40.6	37.2	21.8
60+	44.0	43.4	24.7
Percent women with 10 or more years schooling			
< 30	41.5	38.5	22.2
30–50	33.7	29.8	19.6
> 50	25.6	22.9	18.6
Percentage of institutional births			
< 60	41.0	33.3	18.9
60–80	40.5	36.9	21.1
80+	32.4	30.5	20.8
Percent children fully Immunized			
< 50	39.2	33.6	19.9
50–70	37.5	34.8	21.3
> 70	31.8	29.5	20.2
Percent Children breastfed and received adequate diet			
10–40	41.7	38.8	21.5
40–70	39.9	35.9	21.1
> 70	37.3	33.3	20.3
Percent households with improved sanitation			
< 30	45.1	43.0	23.4
30–50	35.8	32.8	20.7
> 50	29.0	24.4	18.3
Percent households with electricity			
25–60	49.5	42.7	19.4
61–90	41.5	38.7	22.2
> 90	31.7	28.6	20.0

### Spatial heterogeneity of stunting, underweight and wasting

Additional file 1 (Appendix 1) presents the univariate Moran’s *I* statistics depicting the extent of spatial autocorrelation of malnutrition and the meso scale predictors. The Moran’s *I* values were 0.65 for stunting, 0.74 for underweight and 0.51 for wasting. All three coefficients were statistically significant. The district level estimates on poverty head count ratio (PHCR) showed Moran’s *I* value of 0.59. Among all the variables including the malnutrition variables, the Moran’s *I* value was highest for underweight followed by improved sanitation and lowest for full immunisation. Based on Moran’s *I* statistics values, we inferred that the pattern of spatial autocorrelation was highest for underweight followed by stunting and wasting in districts of India. This confirmed the clustering of districts in terms of malnutrition among the children under age five.

**Map 2:**
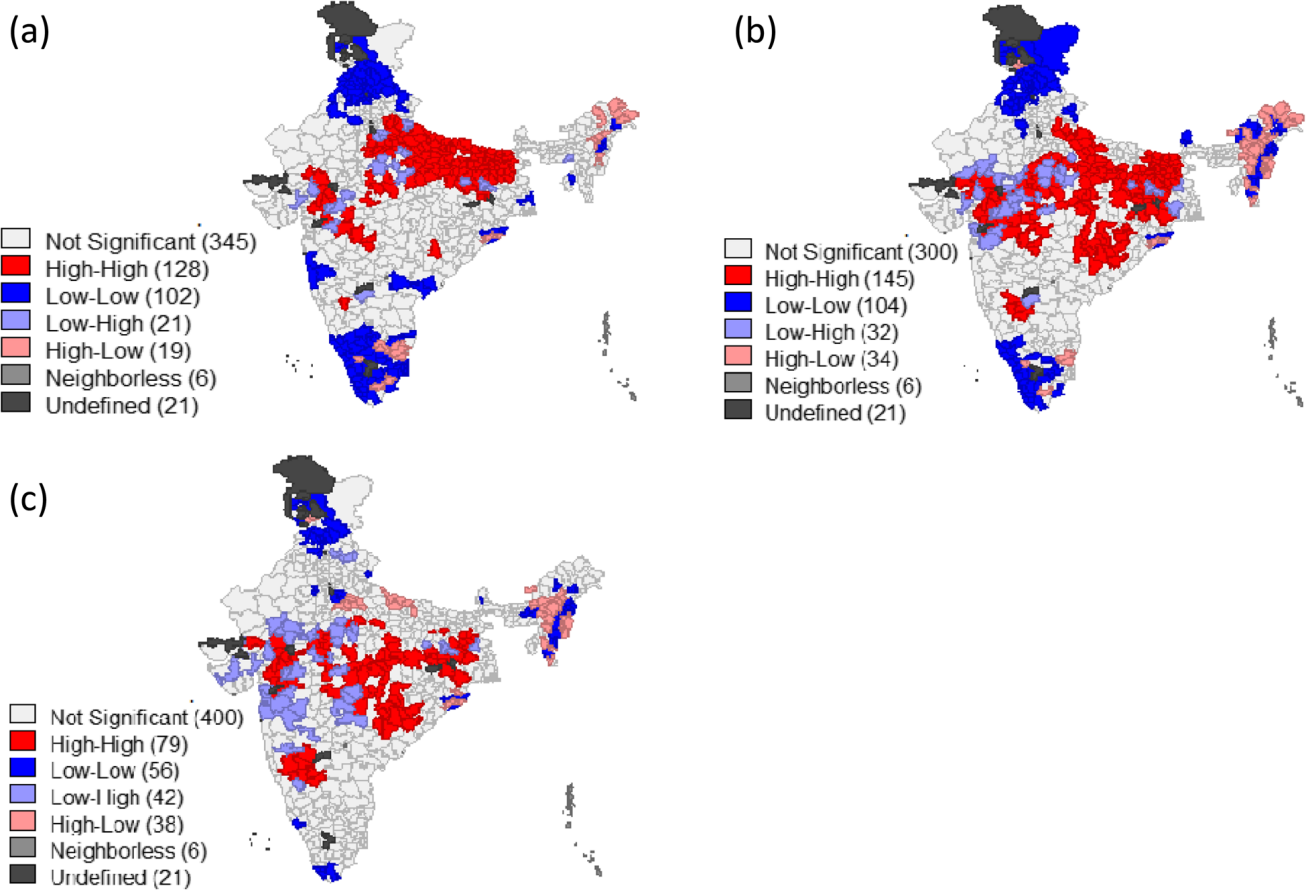
Bivariate LISA cluster maps of India showing the geographic clustering (hotspots & coldspots) of (**a**) poverty vs stunting (**b**) poverty vs underweight (**c**) poverty vs wasting across districts of India, 2015-16. Source: Authors generated the maps using GeoDa version 1.6.7

Table 3 presents the values of Bivariate Moran’s *I* statistics for stunting, underweight and wasting against the correlates. It was found that the spatial autocorrelation of stunting and poverty was 0.46 and that with BMI was 0.53. Similarly, the spatial autocorrelation of underweight and stunting with BMI was 0.66 and 0.52 respectively. In general, poverty and women’s BMI showed high and positive spatial autocorrelation with each of the three measures of malnutrition while women’s education, institutional delivery, children’s immunization status, feeding practices and household level sanitation showed a negative spatial autocorrelation with stunting and underweight. Map 2(a) & (b) present the bivariate LISA cluster map which indicated that about126 of 640 districts (20% of all the districts) had highest prevalence of stunting and highest level of poverty while a cluster of 146 districts (23% of all the districts) were observed as of high underweight and high poverty. These districts were mostly from the states of Uttar Pradesh, Bihar, Jharkhand, Madhya Pradesh, Rajasthan, Maharashtra and Gujarat. A total of 104 districts were classified as cold spots (defined as low poverty and low stunting) from the states of Tamil Nadu, Kerala, Punjab, Himachal Pradesh and some districts of Jammu & Kashmir and Nagaland. Similarly, the hot and cold spots had been identified for underweight and wasted prevalence due to poverty across India and were shown in Map 2(b) and (c).

**Table 3:** Moran’s I Statistics showing the spatial dependence for stunting, underweight and wasted and its correlates in the districts of India, 2015–16

Meso scale variables (district level percentages)	Stunting		Underweight		Wasted	
	Bivariate LISA	Z value	Bivariate LISA	Z value	Bivariate LISA	Z value
Women whose BMI is below 18.5 kg/m^2^	0.52	23.77	0.66	29.38	0.42	20.79
Poverty Head Count ratio	0.46	20.89	0.45	20.12	0.19	9.73
women with 10 or more years schooling	−0.47	−23.19	−0.430	−21.07	−0.18	−9.43
Institutional births	− 0.30	−14.28	− 0.10	−4.84	0.01	5.38
Children fully Immunized	−0.27	−13.43	− 0.14	−7.00	0.00	− 0.04
Children breastfed & received adequate diet	−0.31	−16.26	− 0.28	−14.67	− 0.06	−3.35
Households with improved sanitation	− 0.52	−25.06	− 0.55	−25.32	−0.26	− 14.03
Households with electricity	−0.51	−24.36	− 0.38	−17.60	− 0.02	− 1.21

**Map 3:**
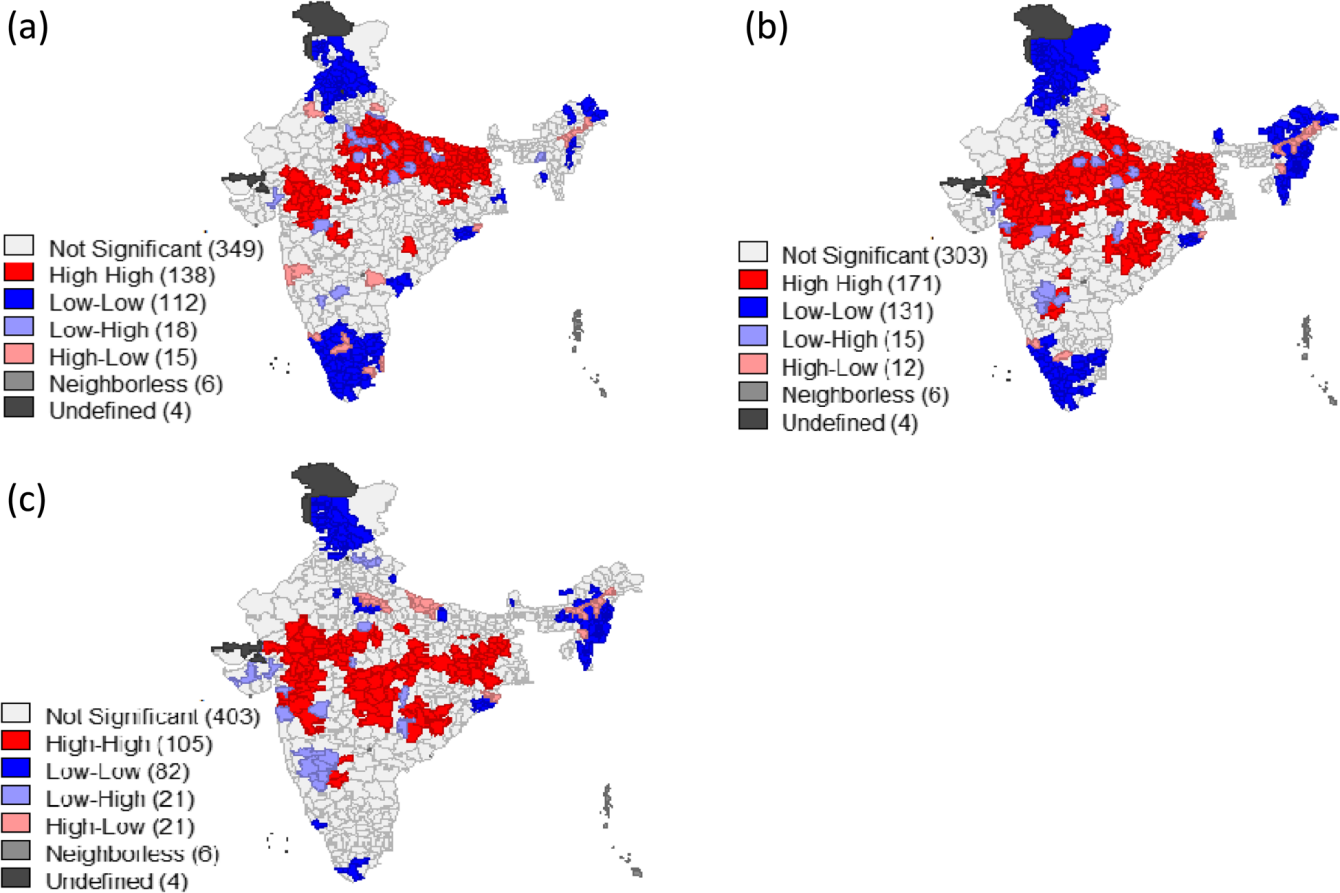
Bivariate LISA cluster maps of India showing the geographic clustering (hotspots & coldspots) of (**a**) bmi of mothers vs stunting (**b**) bmi of mothers vs underweight (**c**) bmi of mothers vs wasting across districts of India, 2015-16. Source: Authors generated the maps using GeoDa version 1.6.7

Map 3 shows the district level clustering of all forms of malnutrition and maternal nutrition considered in terms of BMI less than 18.5 kg/m^2^. The bivariate LISA cluster map suggested that around 135 districts constitute the hot spots (high proportion of low BMI women and high prevalence of stunting) whereas 112 districts constitute the cold spots (low proportion of low BMI women and low prevalence of stunting) (Map 3 (a)). Similarly Map 3 (b) and (c) showed the hot spots for underweight and wasting with BMI. Likewise, the hot and cold spots were identified for mother’s education and sanitation with each of the indicators of malnutrition (Additional file 1: Appendix 3).

### Global spatial regression model

Table 4 presents the results of OLS estimation for stunting, underweight and wasting. OLS estimation showed the preliminary check of the association between malnutrition and the meso scale correlates without considering the spatial structure of the data. From the regression result it was confirmed that BMI status of the mothers and poverty situation across the districts closely determine all the forms of malnutrition (stunting, underweight and wasting). Among the other meso scale correlates mother’s education, breastfeeding pattern and sanitation condition found to be the statistically significant predictors of stunting and underweight prevalence across the districts. After diagnosing the OLS model we found that the residuals in the OLS model were spatially auto-correlated (Stunting: Moran’s *I* = 0.31, *p* value = 0.000001; Underweight: Moran’s *I* = 0.50, *p* value = 0.000001; Wasted: Moran’s *I* = 0.37, *p* value = 0.000001). This suggested that the prevalence of malnutrition among the children was not distributed uniformly across the districts of India and occurred in particular clusters. Hence, we rejected the null hypothesis and accepted that there is a positive spatial autocorrelation in the prevalence of malnutrition and we further estimated the spatial autoregressive models to consider the autocorrelation into account. Spatial lag and error models were fitted in the data. Based upon the model diagnostics we found the spatial error model to be better performing and hence the error model gave the final estimates of the association. Additional file 1: Appendix 3 gives the estimated results from the spatial lag model for all the three indicators of malnutrition. This model estimates confirmed that maternal BMI status and poverty remained the statistically significant predictors of malnutrition from the OLS estimation.

**Table 4:** Result of regression analysis (OLS) showing the adjusted coefficients of the correlates for stunting, underweight and wasted in India, 2015–16

District level meso scale correlates	Stunting		Underweight		Wasted	
	Coef. (95% CI)	*p*-value	Coef. (95% CI)	*p*-value	Coef. (95% CI)	*p*-value
Percent women with below normal BMI	0.30 (0.21,0.39)	0.000	0.72 (0.62,0.81)	0.000	0.39 (0.30,0.49)	0.000
Poverty Head Count ratio	0.07 (0.04,0.11)	0.000	0.10 (0.06,0.14)	0.000	0.07 (0.03, 0.11)	0.001
Percent women (10 or more years education)	−0.05 (−0.11,0.00)	0.062	0.00 (−0.05,0.06)	0.885	0.04 (−0.02, 0.10)	0.213
Percentage of institutional births	−0.09 (− 0.13,-0.05)	0.000	− 0.01 (− 0.06,0.03)	0.523	0.02 (− 0.02, 0.06)	0.368
Percent children fully Immunized	− 0.02 (− 0.05,0.01)	0.230	0.03 (0.00, 0.07)	0.058	0.01 (− 0.02, 0.05)	0.561
Children breastfed & received adequate diet	−0.18 (− 0.25,-0.11)	0.000	− 0.23 (− 0.31, − 0.16)	0.000	−0.08 (− 0.15, 0.00)	0.057
Households with improved sanitation (%)	−0.05 (− 0.08,-0.01)	0.019	−0.08 (− 0.12,-0.04)	0.000	−0.03 (− 0.07, 0.01)	0.132
Households with electricity (%)	−0.13 (− 0.18,-0.08)	0.000	0.01 (− 0.04,0.06)	0.623	0.14 (0.09, 0.19)	0.000
R^2^ Value	0.63		0.69		0.27	
No of districts	640		640		640	

The SEM model gave the final and the spatial endogeneity corrected estimates of the correlates for stunting, underweight and wasted prevalence across India (Table 5). Among the two estimated spatial models, the SEM model showed a lower AIC value for all three nutritional indicators. The results from SEM model are described for each of the nutritional outcome. With respect to stunting, the coefficient was largest for BMI (β = 0.23, 95% CI: 0.14, 0.33) followed by institutional birth (β = − 0.13, 95% CI: -0.18, − 0.08) and mother’s educational attainment (β = − 0.10, 95% CI: -0.17,-0.03). The other significant predictors were poverty and sanitation. Thus the coefficient estimate for BMI confirmed that a 10 point increase in the proportion of women with their BMI below normal across the districts was associated with 2.3 point increase in the stunting prevalence. Similarly, a 10 point increase in the institutional births across the districts was associated with 1.3 point decrease in the stunting prevalence whereas a 10 point increase in the proportion of the educated mothers across districts was statistically supposed to bring down the stunting prevalence by 1 point. On the other hand, poverty and sanitation estimates suggested that a 10 point increase in terms of PHCR was supposed to increase stunting prevalence by 0.7 point whereas sanitation condition (proportion of households with improved sanitation in a district) improvement (10 point) across the districts was found to be associated with decreasing stunting prevalence (by 0.6 point). The corresponding value of the lag coefficient from the stunting model was 0.6 (*p*-value< 0.001). It was observed that, compared the other models, the stunting model showed a lower AIC value with a pseudo R square value of 0.72 indicating the explained variability of the model.

**Table 5:** Estimated results from the Spatial error model for stunting, underweight and wasted in districts of India, 2015–16

District level meso scale correlates	Stunting		Underweight		Wasted	
	Coef. (95% CI)	*p*-value	Coef. (95% CI)	*p*-value	Coef. (95% CI)	*p*-value
Percent women with below normal BMI	0.23 (0.14,0.33)	0.000	0.38 (0.29,0.48)	0.000	0.11 (0.01,0.22)	0.036
Poverty Head Count ratio	0.07 (0.03,0.10)	0.000	0.10 (0.06,0.13)	0.000	0.08 (0.04,0.12)	0.000
Percent women (10 or more years education)	−0.10 (−0.17,-0.03)	0.003	−0.06 (−0.13,0.02)	0.123	0.02 (−0.06,0.09)	0.668
Percentage of Institutional births	−0.13 (− 0.18,-0.08)	0.000	− 0.07 (− 0.12,− 0.02)	0.004	0.00 (− 0.05,0.05)	0.974
Percent children fully Immunized	0.01 (− 0.03,0.05)	0.567	0.05 (0.02,0.09)	0.004	0.02 (− 0.02,0.07)	0.224
Percent children breastfed & received adequate diet	-0.02 (−0.05,0.02)	0.384	−0.03 (− 0.07, 0.01)	0.120	− 0.02 (− 0.06, 0.02)	0.224
Percent households with improved sanitation	− 0.06 (− 0.11, − 0.01)	0.006	−0.06 (− 0.11,-0.01)	0.011	−0.03 (− 0.08,0.02)	0.287
Households with electricity (%)	−0.05 (− 0.11, 0.00)	0.051	−0.01 (− 0.07, 0.04)	0.687	0.04 (− 0.02, 0.10)	0.211
Lambda Value (Lag coefficient)	0.6	0.000	0.75	0.000	0.62	0.000
AIC value	3837		3845		3946	
Pseudo R Square	0.72		0.81		0.45	
No of districts	640		640		640	

The underweight model suggested that, BMI and poverty were two most closely associated predictors of underweight prevalence across the districts. The corresponding coefficients of BMI and poverty were observed to be 0.38 (95% CI: 0.29, 0.48) and 0.10 (95% CI: 0.06, 0.13) respectively. Contrary to the stunting model, maternal education did not seem to be statistically significant predictor for underweight. Among the other correlates, full immunization, institutional births and sanitation appeared to be the significant predictors of underweight. The underweight model suggested a lag of 0.75 and the corresponding pseudo R square value of the model was 0.81 with a slightly higher AIC value (3845) than the stunting model. From the wasted model we found that the district level prevalence of wasted children under age five was mostly determined by maternal nutrition (BMI status) and poverty situation in the districts. The corresponding association between BMI and wasted was 0.11 (*p*-value = 0.036; 95% CI: 0.01, 0.22) whereas this was 0.08 (p-value< 0.001; 95% CI: 0.04, 0.12) for poverty.

Finally, the highly significant error lag value from the estimated SEM models for each of the malnutrition prevalence (stunting, wasting and underweight) indicated that any shock in the omitted variables which were not present but may affect the prevalence of malnutrition was strongly likely to cause a change in the prevalence of malnutrition in the neighbouring districts.

## Discussion

The following are the salient findings from the study. First, the study findings suggest a clear spatial pattern of stunting, underweight and wasting across the districts of India. The Moran’s *I* statistics suggested the measure of spatial dependence and it was found to be highest for stunting followed by underweight and wasting which confirmed the geographical gradient of malnutrition in India. Though a high level of malnutrition spread was found across some selected states and geographical regions, but the clustering was higher in the districts belonging to the states of Uttar Pradesh, Bihar, Jharkhand, Madhya Pradesh and Rajasthan. Second, the spatial analyses suggested statistically significant association of malnutrition (stunting, underweight and wasting) with the factors such as BMI status of mothers, poverty, maternal education and improved sanitation. Study results confirmed that districts with higher incidence of poverty are at higher risk of increased prevalence of stunting, underweight and wasting. Though poverty and malnutrition nexus is complex in different country settings but our results confirmed that poverty significantly affects nutritional status among the children in districts of India. Previous studies for the developing countries proved that poverty is a major contributor to the burden of child malnutrition [44]. Similar to those studies this study also found the poverty, malnutrition linkages and identified districts with high incidence of stunting and poverty, underweight and poverty and wasted and poverty. Prioritising these districts to reduce malnutrition would be helpful to the overall burden of malnutrition in India. The pattern was similar with improved sanitation of the households but of lesser degree. Districts where more proportion of households were availing the facility of improved sanitation showed a lower prevalence of malnutrition across those districts of India. As we know that lack of improved sanitation in the households may lead to childhood diseases such as diarrhoea and other infectious diseases [27, 45]. Parallel to the sanitation argument of child nutrition, it could be mentioned here that Government of India had started a cleanliness program through “Swachh Bharat Abhiyan” to improve sanitation condition and waste management across India [46]. Third, women’s BMI and educational status were found to have positive and strong association with all three nutritional indicators. Districts with higher percentage of women with a BMI less than 18.5 kg/m^2^ were significantly more likely to have higher prevalence of malnutrition among the children and findings were consistent with previous studies. It is well established that maternal nutrition is an important risk factor of poor intrauterine growth and low birth weight during pregnancy and undernourished mothers cannot breast-feed their children adequately causing poor nutrition to their children [28, 29, 47]. Hence, improving maternal health is a prerequisite to reduction of malnutrition among children. In this direction, districts where children were breastfed and received adequate diet were found to be less likely to be burdened with stunting. Breastfeeding pattern and initiation of complementary feeding and quality of the complementary food could be the possible reason which helped the prevalence of child malnutrition to reduce in those districts [48, 49].

Similarly, malnutrition was found to be negatively and significantly associated with women’s educational attainment. Our findings also support the positive linkage between women’s educational attainment and child’s nutrition. This finding is also consistent with the previous studies which establish the impact of mother’s education on child’s nutritional status [50, 51]. Fourth, mapping of districts identified the hot-spot and cold-spot based on clustering of malnutrition with the meso scale correlates at district level which would be helpful to the planners and policy makers to help build new intervention for those specific underprivileged districts. In this context, the National Health Mission (NHM) initiative by Govt. of India is working towards child and maternal health across India to improve over the situation prevailing. In another public health intervention to fight against malnutrition, Govt of India has set up the National Nutrition Mission (NNM) for programmatic intervention with a three year budget of Rs.9046.17 crore which was commenced in 2017 in the high priority districts [52, 53].

## Conclusion

This study illustrates the spatial heterogeneity of malnutrition among the children in districts of India. The findings could be useful for public health planning and targeting the underlying meso scale factors associated with child nutrition in India. It also suggests allocation of health resources and the implementation of child health specific interventions in the geographical hotspots of higher malnutrition prevalence. The spatial clustering of malnutrition is found in those geographical pockets where poverty is high, women’s education is low, BMI level among women is below normal. The malnutriton indicators also  significantly falls in terms of other distal and proximate factors, which reinforces the need for intersectoral co-ordination in fighting malnutrition in India. An integrated approach; a multisectoral co-ordination of reduction of poverty, increasing sanitation coverage and maternal nutrition can help to reduce child malnutrition in India.

## Additional file

Additional file 1: **Appendix 1.** Moran’s I Statistics showing the spatial dependence for the district level prevalence of stunting and the meso scale indicators in India, 2015–16. **Appendix 2.** Estimated results from Spatial lag model for stunting, underweight and wasted, India, 2015–16. **Appendix 3.** Bivariate LISA cluster maps, India, 2015–16. **Appendix 4.** Districts with higher prevalence of stunting (46–65)% among children under age five, India, 2015–16. **Appendix 5.** Districts with higher prevalence of underweight (44–67)% among children under age five, India, 2015–16. **Appendix 6.** Districts with higher prevalence of wasting (28–47)% among children under age five, India, 2015–16 (PDF 192 kb).

## Abbreviations

AIC: Akaike information criterion
AIDS: Acquired Immune Deficiency Syndrome
ARI: Acute respiratory infection
BMI: Body mass index
CFP: Child feeding practice
GDP: Gross Domestic Product
HAZ: Height-for-age z score
LISA: Local Indicator of Spatial Association
MDG: Millennium development goal
NFHS: National Family Health Survey
OLS: Ordinary least square
PHCR: Poverty head count ratio
SDG: Sustainable development goal
SEM: Spatial error model
SLM: Spatial lag model
WAZ: Weight-for- age z score
WHZ: Weight-for-height z score
