# Supplementary material for: Spatial heterogeneity and correlates of child malnutrition in districts of India
Source: BMC Public Health. 2018 Aug 17;18:1027. doi: 10.1186/s12889-018-5873-z (PMC6098604; doi:10.1186/s12889-018-5873-z)
Supplement: Supplementary file 1 — Appendix 1. Moran’s I Statistics showing the spatial dependence for the district level prevalence of stunting and the meso scale indicators in India, 2015–16. Appendix 2. Estimated results from Spatial lag model for stunting, underweight and wasted, India, 2015–16. Appendix 3. Bivariate LISA cluster maps, India, 2015–16. Appendix 4. Districts with higher prevalence of stunting (46–65)% among children under age five, India, 2015–16. Appendix 5. Districts with higher prevalence of underweight (44–67)% among children under age five, India, 2015–16. Appendix 6. Districts with higher prevalence of wasting (28–47)% among children under age five, India, 2015–16 (PDF 192 kb). [file 12889_2018_5873_MOESM1_ESM.pdf]

Appendix 1: Moran's I Statistics showing the spatial dependence for the district level prevalence of stunting and the meso scale indicators in India, 2015-16

District level Meso scale Indicators/Variables	Moran's I values	Z value
Stunting	0.65	25.13
Underweight	0.74	29.91
Wasted	0.51	20.1
Women whose BMI is below normal	0.69	27.13
Poverty Head Count ratio	0.59	21.71
women with 10 or more years schooling	0.69	27.66
Institutional births	0.66	23.95
Children fully Immunized	0.55	20.24
Children breastfed & received adequate diet	0.64	25.56
Households with improved sanitation	0.74	30.3
Households with electricity	0.70	23.35

Appendix 2: Estimated results from Spatial lag model for stunting, underweight and wasted, India, 2015-16.

	Stunting		Underweight		Wasted	
District level meso scale correlates	Coef. (95% CI)	p-value	Coef. (95% CI)	p-value	Coef. (95% CI)	p-value
Percent women whose BMI is below 18.5kg/m^2	0.21 (0.13,0.29)	0.000	0.44 (0.36,0.52)	0.000	0.2 (0.12,0.28)	0.000
Poverty Head Count ratio	0.06 (0.02,0.09)	0.001	0.07(0.03,0.10)	0.000	0.05 (0.02,0.09)	0.005
Percent women (10 or more years education)	-0.07 (-0.12,-0.02)	0.005	-0.03 (-0.08,0.02)	0.299	0.02 (-0.04,0.07)	0.516
Percentage of Institutional births	-0.07 (-0.10,-0.03)	0.000	-0.01 (-0.05,0.02)	0.501	0.01 (-0.02,0.05)	0.493
Percent children fully Immunized	-0.02 (-0.05,0.01)	0.265	0.03 (0.00,0.06)	0.055	0.01 (-0.02,0.04)	0.422
Percent children breastfed & received adequate diet	-0.04(-0.07,-0.01)	0.006	-0.04 (-0.07,-0.01)	0.006	-0.01 (-0.04,0.02)	0.500
Percent households with improved sanitation	-0.03 (-0.06,0.01)	0.149	-0.03 (-0.06,0.01)	0.114	-0.01 (-0.05,0.03)	0.559
Households with electricity (%)	-0.06 (-0.11,-0.02)	0.004	0.01 (-0.03,0.05)	0.612	0.07 (0.02,0.12)	0.003
Rho Value (Lag coefficient)	0.362		0.495		0.501	
AIC value	3870.54		3866.75		3947.42	
Pseudo R Square	0.6924		0.7848		0.4292	
No of districts	640		640		640	

Appendix 3: Bivariate LISA cluster maps, India, 2015-16

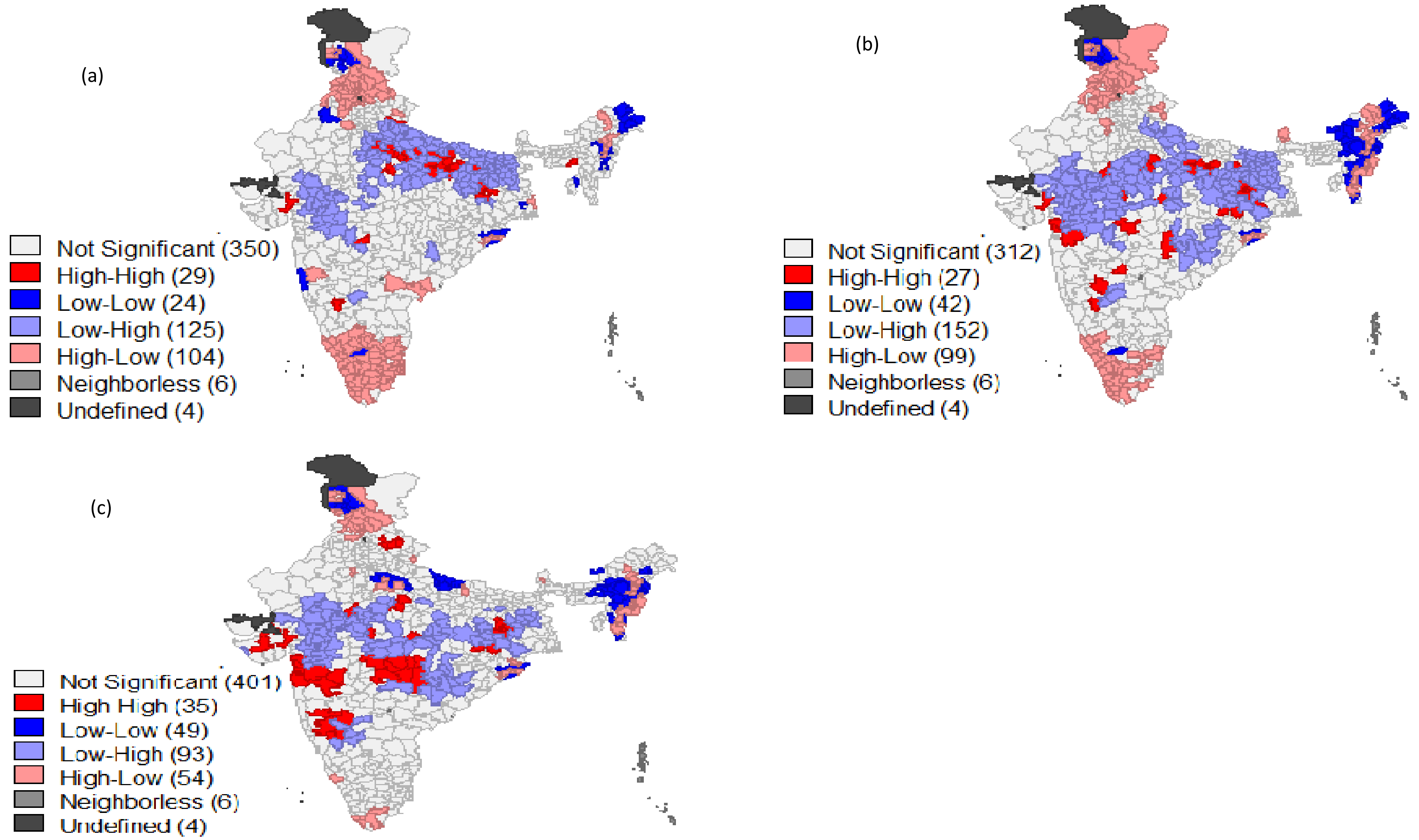

Map 1. Bivariate LISA cluster maps of India visualising the geographic clustering (hotspots & coldspots) of (a) education vs stunting (b) education vs underweight (c) education vs wasting across districts of India, 2015-16. Source: Authors generated the maps using GeoDa version 1.6.7

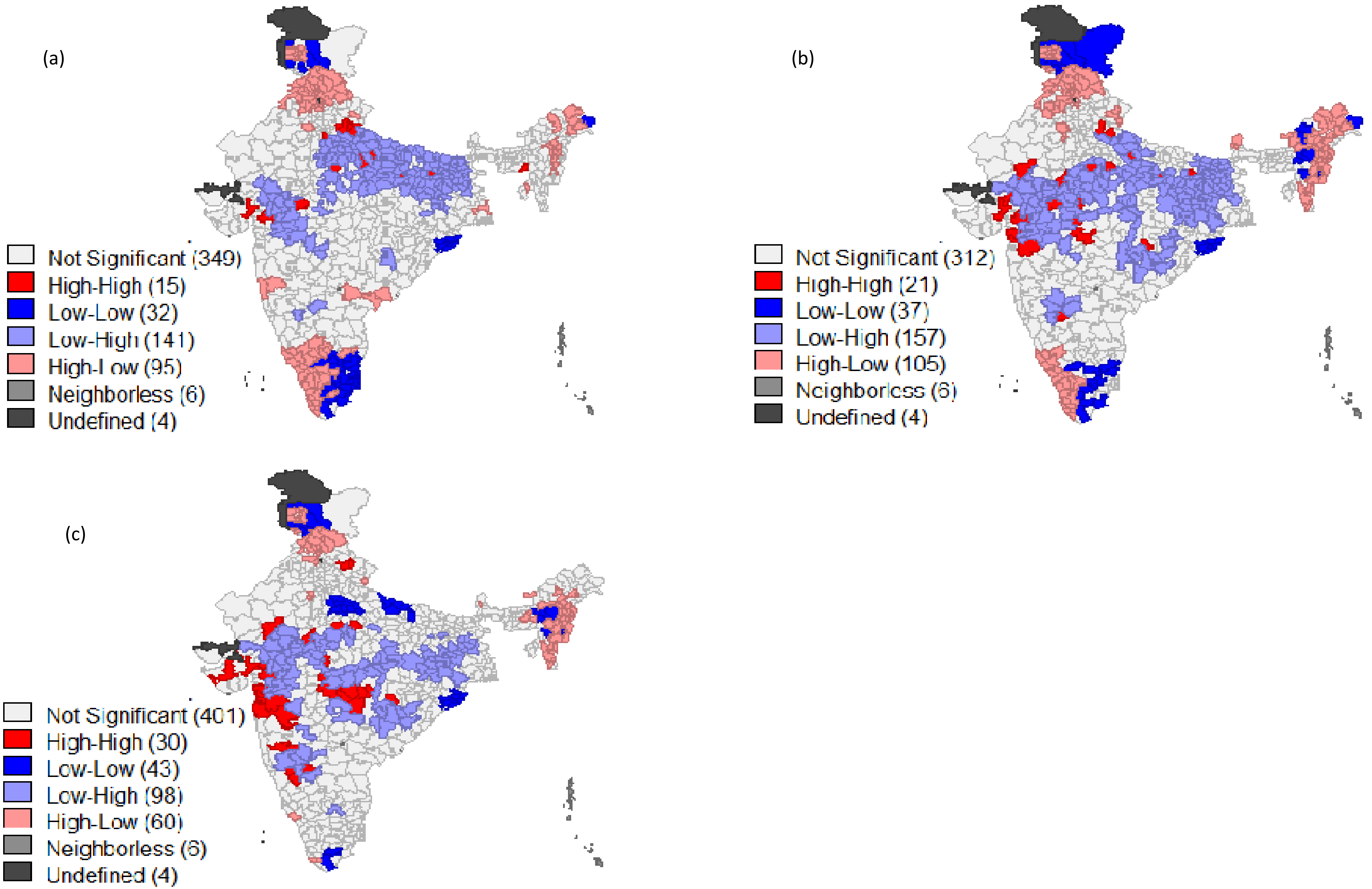

Map 2. Bivariate LISA cluster maps of India visualising the geographic clustering (hotspots & coldspots) of (a) sanitation vs stunting (b) sanitation vs underweight (c) sanitation vs wasting across districts of India, 2015-16. Source: Authors generated the maps using GeoDa version 1.6.7

Appendix 4: Districts with higher prevalence of stunting (46-65)% among children under age five, India, 2015-16

State	Districts						
Bihar	Banka	Katihar	Araria	Darbhanga	Madhepura	Munger	Aurangabad
	Madhubani	Samastipur	Bhagalpur	Kishanganj	Arwal	Khagaria	Jehanabad
	Gaya	Sitamarhi	Purba Champaran	Nawada	Rohtas	Muzaffarpur	Vaishali
	Nalanda	Lakhisarai	Purnia	Sheohar	Sheikhpura	Kaimur (Bhabua)	
	Supaul						
Uttar Pradesh	Fatehpur	Shrawasti	Kheri	Unnao	Farrukhabad	Bara Banki	Mainpuri
	Mahrajganj	Kanshiram Nagar	Basti	Etawah	Shahjahanpur	Pilibhit	Banda
	Sant Kabir Nagar	Sitapur	Chitrakoot	Sant Ravidas Nagar (Bhadohi)	Gonda	Faizabad	Bahraich
	Balrampur	Aligarh	Siddharthnagar	Hardoi	Budaun	Mirzapur	Jaunpur
	Kaushambi	Kannauj	Etah				
Madhya Pradesh	Datia	Sheopur	Morena	Alirajpur	Bhind	Sidhi	Shajapur
	Burhanpur	Khargone (West Nimar)	Bhopal	Barwani	Tikamgarh	Shivpuri	
Rajasthan	Udaipur	Bharatpur	Banswara	Dhaulpur	Dungarpur	Pratapgarh	
Karnataka	Yadgir	Gulbarga	Bagalkot	Bellary	Davanagere	Koppal	
Jharkhand	Pakur	Hazaribagh	Chatra	Godda	Sahibganj	Pashchimi Singhbhum	
Gujarat	Narmada	Sabar Kantha	The Dangs	Anand	Bhavnagar		
Chhattisgarh	Rajnandgaon	Bijapur	Narayanpur				
Meghalaya	Jaintia Hills	East Khasi Hills	Ribhoi	West Khasi Hills			
Maharashtra	Yavatmal	Nandurbar	Parbhani				
Haryana	Mewat						
Assam	Dhubri						
Odisha	Subarnapur						

Appendix 5: Districts with higher prevalence of underweight (44-67)% among children under age five, India, 2015-16

States	Districts						
Bihar	Jamui	Banka	Saharsa	Katihar	Araria	Madhepura	Aurangabad
	Nalanda	Madhubani	Kishanganj	Arwal	Jehanabad	Lakhisarai	Sheikhpura
	Gaya	Sitamarhi	Nawada	Rohtas	Bhojpur	Purnia	
	Kaimur (Bhabua)						
Uttar Pradesh	Rampur	Lalitpur	Sonbhadra	Budaun	Kaushambi	Lucknow	Jalaun
	Mahoba	Shahjahanpur	Pilibhit	Auraiya	Varanasi	Mirzapur	Sitapur
	Chitrakoot	Sant Ravidas Nagar (Bhadohi)	Faizabad	Jaunpur	Tehri Garhwal	Puruliya	
Madhya Pradesh	Datia	Mandla	Sheopur	Morena	Alirajpur	Raisen	Bhind
	Gwalior	Rajgarh	Shajapur	Dhar	Betul	Barwani	Ashoknagar
	Umaria	Khandwa (East Nimar)	Burhanpur	Dewas	Khargone (West Nimar)	Dindori	
	Guna	Shivpuri					
Jharkhand	Purbi Singhbhum	Pakur	Simdega	Deoghar	Latehar	Gumla	Jamtara
	Lohardaga	Hazaribagh	Khunti	Chatra	Dumka	Garhwa	Bokaro
	Saraikela-Kharsawan	Sahibganj	Ramgarh		Godda		
Gujarat	Narmada	Dohad	Surendranagar	Kheda	The Dangs	Bharuch	Bhavnagar
	Sabar Kantha						
Odisha	Kendujhar	Sundargarh	Nabarangapur	Balangir	Malkangiri	Sambalpur	Koraput
Rajasthan	Udaipur	Banswara	Jhalawar	Dungarpur	Pratapgarh	Sirohi	
Karnataka	Yadgir	Gulbarga	Bagalkot	Bellary	Koppal		
Chhattisgarh	Bastar	Bijapur	Narayanpur	Uttar Bastar Kanker	Dakshin Bastar Dantewada		
Maharashtra	Osmanabad	Yavatmal	Dhule	Nandurbar			
Uttarakhand	Tehri Garhwal						
West Bengal	Puruliya						

Appendix 6: Districts with higher prevalence of wasting (28-47)% among children under age five, India, 2015-16

States	Districts						
Jharkhand	Purbi Singhbhum	Pashchimi Singhbhum	Latehar	Gumla	Dhanbad	Jamtara	Lohardaga
	Khunti	Chatra	Dumka	Garhwa	Bokaro	Simdega	Ramgarh
Maharashtra	Washim	Gondiya	Bid	Yavatmal	Dhule	Raigarh	Thane
	Chandrapur	Gadchiroli	Jalgaon	Nashik	Nandurbar		
Madhya Pradesh	Mandla	Sheopur	Morena	Alirajpur	Bhind	Anuppur	Rajgarh
	Shajapur	Seoni	Dhar	Betul	Barwani	Ashoknagar	Balaghat
	Chhindwara	Singrauli	Jabalpur	Hoshangabad	Guna		
Rajasthan	Ajmer	Udaipur	Banswara	Baran	Jhalawar	Rajsamand	Dungarpur
	Bhilwara	Pratapgarh	Sirohi				
Gujarat	Narmada	Gandhinagar	The Dangs	Tapi	Panch Mahals	Jamnagar	Bharuch
	Valsad	Junagadh	Kachchh				
Odisha	Sundargarh	Nabarangapur	Malkangiri	Sambalpur	Koraput		
Uttar Pradesh	Lalitpur	Rae Bareli	Kaushambi	Lucknow	Jalaun	Hamirpur	Chitrakoot
Karnataka	Yadgir	Gulbarga	Bangalore	Belgaum	Bijapur	Chitradurga	Gadag
	Dharwad	Raichur					
Chhattisgarh	Bastar	Koriya	Narayanpur	Uttar Bastar Kanker	Dakshin Bastar Dantewada		
Tamil Nadu	Dharmapuri	The Nilgiris	Tiruvannamalai				
Bihar	Arwal	Sheikhpura	Patna				
Uttarakhand	Dehradun	Uttarkashi					
West Bengal	Puruliya	Birbhum					
Haryana	Ambala	Panchkula					
Andaman & Nicobar Islands	North & Middle Andaman						
Arunachal Pradesh	Upper Siang						
Meghalaya	South Garo Hills						
